# Antimicrobial Resistance, Virulence Profiles and Molecular Subtypes of *Salmonella enterica* Serovars Typhi and Paratyphi A Blood Isolates from Kolkata, India during 2009-2013

**DOI:** 10.1371/journal.pone.0101347

**Published:** 2014-08-06

**Authors:** Shanta Dutta, Surojit Das, Utpala Mitra, Priyanka Jain, Indranil Roy, Shelley S. Ganguly, Ujjwayini Ray, Phalguni Dutta, Dilip Kumar Paul

**Affiliations:** 1 National Institute of Cholera and Enteric Diseases, Kolkata, India; 2 Calcutta Medical Research Institute, Kolkata, India; 3 Advance Medical Research Institute, Salt Lake, Kolkata, India; 4 Apollo Gleneagles Hospitals, Kolkata, India; 5 Dr. B. C. Roy Post Graduate Institute of Pediatric Sciences, Kolkata, India; New York State Dept. Health, United States of America

## Abstract

Enteric fever, caused by *Salmonella enterica,* remains an unresolved public health problem in India and antimicrobial therapy is the main mode of treatment. The objective of this study was to characterize the *Salmonella enterica* isolates from Kolkata with respect to their antimicrobial resistance (AMR), virulence profiles and molecular subtypes. *Salmonella enterica* blood isolates were collected from clinically suspected enteric fever patients attending various hospitals in Kolkata, India from January 2009 to June 2013 and were tested for AMR profiles by standard protocols; for resistance gene transfer by conjugation; for resistance and virulence genes profiles by PCR; and for molecular subtypes by Pulsed Field Gel Electrophoresis (PFGE). A total of 77 *Salmonella enterica* serovar Typhi (*S*. Typhi) and 25 *Salmonella enterica* serovar Paratyphi A (*S*. Paratyphi A) from Kolkata were included in this study. Although multidrug resistance (resistance to chloramphenicol, ampicillin, co-trimoxazole) was decreasing in *S*. Typhi (18.2%) and absent in *S.* Paratyphi A, increased resistance to fluoroquinolone, the current drug of choice, caused growing concern for typhoid treatment. A single, non-conjugative non-IncHI1 plasmid of 180 kb was found in 71.4% multidrug resistant (MDR) *S*. Typhi; the remaining 28.6% isolates were without plasmid. Various AMR markers (*bla*_TEM-1_, *cat*A, *sul*1, *sul*2, *dfr*A15, *str*A-*str*B) and class 1 integron with *dfr*A7 gene were detected in MDR *S*. Typhi by PCR and sequencing. Most of the study isolates were likely to be virulent due to the presence of virulence markers. Major diversity was not noticed among *S*. Typhi and *S*. Paratyphi A from Kolkata by PFGE. The observed association between AMR profiles and *S*. Typhi pulsotypes might be useful in controlling the spread of the organism by appropriate intervention. The study reiterated the importance of continuous monitoring of AMR and molecular subtypes of *Salmonella* isolates from endemic regions for better understanding of the disease epidemiology.

## Introduction

Enteric fever caused by *Salmonella enterica* serovar Typhi (*S*. Typhi) and *Salmonella enterica* serovar Paratyphi A (*S*. Paratyphi A) still continues to be a major public health problem in developing countries like India. Typhoid fever was estimated to have caused 21.6 million illnesses and 216,500 deaths globally, and the less severe paratyphoid fever caused an estimated 5.4 million illnesses in 2000 [1]. The incidence of typhoid fever was estimated as 214.2 per 100,000 people/year during 2003–2004 in a population-based study conducted at different urban slums of Kolkata [2]. Although *S*. Typhi was observed to be the predominant serovar worldwide, a shift in the most prevalent *Salmonella* serotype from *S*. Typhi to *S*. Paratyphi A has been reported recently by many researchers [3]–[6].

Antimicrobial therapy is the mainstay for treatment of enteric fever and typhoid fever may become fatal in 30% cases due to complications in absence of appropriate antibiotics. Since the late eighties, increased isolation of multidrug resistant (MDR, resistant to ampicillin, chloramphenicol and co-trimoxazole with or without tetracycline) *S*. Typhi was reported from different parts of India limiting the use of these drugs in the treatment of typhoid [7], [8]. Subsequently ciprofloxacin was used as the drug of choice in adults. But frequent use of ciprofloxacin led to the global emergence of nalidixic acid resistant *S*. Typhi and *S*. Paratyphi A associated with decreased susceptibility to ciprofloxacin, causing increased treatment failure cases [6], [9]–[12]. Thereafter emergence of ciprofloxacin resistant *S*. Typhi and *S*. Paratyphi A further decreased the number of treatment options [10], [13], [14]. Sporadic reports of *Salmonella enterica* isolates resistant to higher generation cephalosporins like ceftazidime, cefotaxime, cefuroxime erased even the last option for treatment of enteric fever [15]–[17]. Azithromycin has been used as an effective alternative in uncomplicated cases, but resistance to this antibiotic has been reported from India and other countries [18 19, 20]. Knowledge on antimicrobial resistance (AMR) profiles of *Salmonella enterica* blood isolates is important for predicting the best possible treatment option for enteric fever patients of any region. Many genes involved in AMR are found on mobile elements [21], [22], and are therefore likely to be transferred between bacterial pathogens, which has a significant public health impact.

*S*. Typhi and *S*. Paratyphi A are intracellular pathogens possessing a myriad of virulence factors which contribute in disease progression and severity. The virulence genes are generally distributed on *Salmonella* pathogenicity islands (SPIs), which are large genomic regions of 3–150 kb acquired horizontally [23]. Virulence gene profiles of the local isolates could shed light on the virulence types of the organisms and predict clinical sequels of the disease.

Molecular typing of any organism is necessary to determine the relatedness among the isolates and to study the molecular epidemiology of the organism. Pulsed Field Gel Electrophoresis (PFGE) is used as a standard and most popular molecular tool for outbreak investigations and tracking the route of transmission [24]. Only a few studies have shown association of AMR profiles and PFGE subtypes of *Salmonella enterica* isolates [5], [25]–[27], but reports are lacking from the Indian subcontinent.

In view of this background information, the present study was undertaken to characterize the *Salmonella enterica* blood isolates from Kolkata during 2009 to 2013 with respect to their AMR profiles, plasmid profiles, virulence genes profiles and PFGE subtypes.

## Materials and Methods

### Ethics statement

The present study was reviewed and approved by the Institutional Ethical Committee of National Institute of Cholera and Enteric Diseases (NICED), Kolkata. Blood samples were collected from febrile children after receiving informed written consent from their parents or guardians on behalf of the children enrolled.

### The study strains

#### Collection of *Salmonella enterica* blood isolates from a hospital based prospective study

Children (<15 years) with clinically diagnosed enteric fever, attending the Out Patient Department (OPD) of Dr. B. C. Roy Post Graduate Institute of Pediatric Sciences, Kolkata, West Bengal during January 2009 to June 2013 were enrolled in this study. Blood samples (3–5 ml) were collected aseptically from the patients, inoculated into BACTEC Peds Plus bottles (BD BACTEC system, Franklin Lakes, NJ, USA) and processed at the microbiology laboratory of NICED, Kolkata for different *Salmonella enterica* serovars. The inoculated bottles were incubated at 37°C for 7 days in a BACTEC 9120 system (BD, Franklin Lakes, NJ, USA) and subcultures were made on MacConkey agar (Difco, Sparks, MD, USA) plates as and when the machine indicated. Non-lactose fermenting smooth colonies were provisionally identified as *Salmonella enterica* by Gram stain and other biochemical tests following standard protocol [28]. Serovar confirmation was done by serological agglutination using *Salmonella* poly- and monovalent antisera (Denka Seiken Co Ltd., Tokyo, Japan). On 7^th^ day of incubation, blood culture was read as negative for *Salmonella*, if there was no visible growth.

#### Collection of *Salmonella enterica* blood isolates from other hospitals in Kolkata

*Salmonella enterica* blood isolates were also collected for the study from the following three other hospitals in Kolkata during the study period: Calcutta Medical Research Institute (CMRI), Apollo Gleneagles Hospital (AGH) and Advance Medical Research Institute (AMRI) located in different parts of Kolkata. All the collected isolates were confirmed as *Salmonella enterica* by microbiological methods as described earlier.

### Antimicrobial susceptibility testing and determination of Minimum Inhibitory Concentration

Antimicrobial susceptibility testing was performed by Kirby Bauer disc diffusion method using the following 17 antimicrobial discs: ampicillin (10 µg), chloramphenicol (30 µg), co-trimoxazole (25 µg), tertracycline (30 µg), streptomycin (10 µg), amoxicillin/clavulanic acid (20/10 µg), amikacin (30 µg), gentamicin (10 µg), nalidixic acid (30 µg), ciprofloxacin (5 µg), ofloxacin (5 µg), levofloxacin (5 µg), ceftriaxone (30 µg), ceftazidime (30 µg), cefotaxime (30 µg), aztreonam (30 µg) and azithromycin (15 µg). Interpretation was done according to the Clinical and Laboratory Standards Institute (CLSI) 2013 guidelines [29]. *Escherichia coli* ATCC 25922 was used as control. The minimum inhibitory concentrations (MICs) of the antibiotics were determined by E-test (AB Biodisk, Solna, Sweden). Decreased susceptibility to ciprofloxacin was defined as isolates having MIC of ciprofloxacin 0.125–0.5 µg/ml. Strains with MIC ≥1 µg/ml were defined as ciprofloxacin resistant. MIC breakpoints of streptomycin and azithromycin were not published for *Enterobacteriaceae* in CLSI 2013. Streptomycin resistance was defined at MIC ≥64 µg/ml according to the US National Antimicrobial Resistance Monitoring System-USDA (http://www.ars.usda.gov/Main/docs.htm?docid=6750). The “epidemiological cutoff” for resistance to azithromycin in *Salmonella* was documented at >16 µg/ml [30].

### Plasmid extraction and incompatibility typing

Extraction of heavy plasmids of all the study isolates was done by Kado and Liu method [31], and *E. coli* V517 and *Shigella flexneri* YSH6000 reference strains were used as molecular weight markers. GenElute Plasmid Miniprep kit (Sigma, St. Louis, MO) was used for extraction of smaller plasmids and supercoiled plasmid DNA (2 to 10 kb) was used as the standard marker. Plasmids were separated by electrophoresis on 0.8% agarose gels and were visualized with a UV-transilluminator after staining the gel with ethidium bromide (0.5 µg/ml). Plasmid incompatibility typing of *S*. Typhi isolates was performed by PCR using published primers of the 18 major incompatibility groups (FIA, FIB, FIC, HI2, I1-Iγ, L/M, N, P, W, T, A/C, K, B/O, X, Y, F, FIIA including IncHI1 type) among *Enterobacteriaceae* [32].

### Determination of antimicrobial resistance gene markers by PCR

MDR *Salmonella* study isolates were screened for the presence of following resistance genes by PCR using published primers (Table 1): *bla*_TEM_, *bla*_SHV_ and *bla*_OXA_ genes for β-lactamase; *cat* gene for chloramphenicol resistance; *sul*1, *sul*2, *sul*3 and *dfr* genes for sulphonamide and trimethoprim resistance; *tet*A and *tet*B for tetracycline resistance; *aad*A, *str*A and *str*B genes for streptomycin resistance and integrase *int*1 gene for the presence of class 1 integron. The gene cassette within the variable region of class 1 integron and the presence of *qacEΔ*1 gene at 3′ end of the conserved segment were also determined [22], [33], [34]. Alleles of *bla*_TEM_, *cat, dfr, aad*A genes and the resistance gene cassettes of class 1 integron were determined by direct sequencing of the PCR products using a 3730 DNA analyzer (Applied Biosystem, Foster City, CA, USA). Sequences obtained were analyzed by comparison with the National Centre for Biotechnology Information (NCBI) database sequences using BLAST program. Suitable positive and negative controls were included in each run.

**Table 1:** Primers list for detection of resistance genes in *S.* Typhi Kolkata isolates.

Resistance Gene	Oligonucleotide sequences (5′→3′)	PCR Product size (bp)	Reference
*bla*_TEM_	TTTTCGTGTCGCCCTTATTCC CGTTCATCCATAGTTGCCTGACTC	798	[22]
*bla*_OXA_	ACACAATACATATCAACTTCGC AGTGTGTTTAGAATGGTGATC	813	[33]
*bla*_SHV_	CACTCAAGGATGTATTGTG TTAGCGTTGCCAGTGCTCG	885	[33]
*cat*	TCCCAATGGCATCGTAAAGAAC TCGTGGTATTCACTCCAGAGCG	293	[22]
*sul*1	TGGTGACGGTGTTCGGCATTC GCGAGGGTTTCCGAGAAGGTG	789	[33]
*sul*2	CGGCATCGTCAACATAACC GTGTGCGGATGAAGTCAG	722	[33]
*sul*3	CATTCTAGAAAACAGTCGTAGTTCG CATCTGCAGCTAACCTAGGGCTTTGGA	990	[33]
*dfr*	GTGAAACTATCACTAATGG TTAACCCTTTTGCCAGATTT	474	[33]
*tet*A	GTAATTCTGAGCACTGTCGC CTGCCTGGACAACATTGCTT	937	[33]
*tet*B	CTCAGTATTCCAAGCCTTTG CTAAGCACTTGTCTCCTGTT	416	[33]
*str*A	CTTGGTGATAACGGCAATTC CCAATCGCAGATAGAAGGC	548	[34]
*str*B	ATCGTCAAGGGATTGAAACC GGATCGTAGAACATATTGGC	509	[34]
*aad*A	GTGGATGGCGGCCTGAAGCC ATTGCCCAGTCGGCAGCG	528	[34]
*intI*1	GGGTCAAGGATCTGGATTTCG ACATGGGTGTAAATCATCGTC	483	[33]
*intI*1 variable region	GGCATCCAAGCAGCAAG AAGCAGACTTGACCTGA	variable	[33]
*qac*EΔ1	GGCTGGCTTTTTCTTGTTATCG TGAGCCCCATACCTACAAAGC	287	[33]

### Conjugation experiment for antimicrobial resistance transfer

We performed conjugation experiments to determine whether antimicrobial resistance markers were located on conjugative plasmids by using wild MDR *Salmonella* isolates as donor and commonly used *E. coli* K-12 MG1655 (susceptible to all antibiotics) as recipient strain [34], [35]. Donor and recipient strains at exponential phase were grown in Trypticase soy broth (Difco, Sparks, MD, USA) and were mixed and incubated at 37°C for 20 h. Transconjugants were selected as lactose fermenting colonies on MacConkey agar plates containing following antibiotics: ampicillin (50 µg/ml), chloramphenicol (50 µg/ml) or tetracycline (16 µg/ml). Antimicrobial susceptibilities of the transconjugants were tested by disc diffusion, MICs by E-test and resistance markers were determined by PCR as stated earlier.

### Detection of virulence gene markers by PCR

A total of 12 virulence markers, located on and outside SPIs were screened by PCR using suitable published primers or primers designed in this study. The virulence genes selected for screening the isolates were *inv*A, *ssa*Q, *spi4*D, *via*B, *stn, hil*A, *ssr*B, *mgt*C, *sop*B, *sci*N, *saf*B and *plt*A [23], [36].

### Pulsed Field Gel Electrophoresis

PFGE was performed following the PulseNet one-day standard protocol [37]. Briefly, bacterial cells were suspended in 1 ml cell suspension buffer (100 mM Tris, 100 mM EDTA, pH 8.0). The cell suspension (200 µl) was mixed with 10 µl proteinase K (20 mg/ml; Sigma) and molten 1% Seakem agarose (200 µl) (Lonza, USA) for preparing solid agarose plugs. The plugs were treated with 5 ml cell lysis buffer (50 mM Tris, 50 mM EDTA, pH 8.0, 1% Sarcosyl) and 25 µl proteinase K (20 mg/ml) and incubated at 54°C for 2 h in a shaking water bath. The plugs were washed thoroughly with pre-warmed water for two times and pre-warmed Tris-EDTA buffer (pH 8.0) for four times at 10 mins interval at 50°C. The DNA plugs were digested with 40 U of *Xba*I (New England Biolabs, MA) at 37°C for 18 h. The digested DNA was run on 1% pulsed field certified agarose gel (Bio-Rad, Hercules, Calif.) prepared in 0.5x TBE buffer (Sigma) using CHEF DRIII (Bio-Rad) apparatus with an initial switch time of 2.2 sec and a final switch time of 63.8 sec at 6 V/cm for 24 h. The gel was stained with ethidium bromide (1 µg/ml, Sigma), destained with deionized water and PFGE profiles were observed with the UV trans-illuminator using GelDoc (Bio-Rad). *Salmonella enterica* serovar Braenderup H9812 was used as reference strain.

### Analysis of PFGE patterns

The PFGE patterns were analyzed using FPQuest software version 4.5 (Bio-Rad) by methods described earlier [38]. Similarity analysis was done by using Dice coefficient with 1.5% optimization and tolerance levels and clustering of matched bands was done using Unweighted Pair Group Method with Arithmetic mean (UPGMA). The isolates with identical PFGE patterns were described as genetically indistinguishable (Dice coefficient of similarity of 100%); isolates with PFGE patterns differing by three or less bands were designated as related (Dice coefficient of similarity of >80%).

## Results

### Isolation of *Salmonella enterica* from prospective samples and collection of strains from other hospitals

A total of 422 hospital (Dr. B. C. Roy Post Graduate Institute of Pediatric Sciences) attending febrile children of which 257 (60.9%) were male and 165 (39.1%) were female with clinical diagnosis of enteric fever were enrolled in the study. Median age of the patients was 5 years (range, 1 month to 15 years) and median duration of fever was 7 days (range, 2 to 90 days). Out of 422 blood samples processed, 34 (8%) were positive for *Salmonella enterica* of which 27 (6.4%) were *S*. Typhi and 7 (1.6%) were *S*. Paratyphi A. Additionally a total of 68 (50 *S*. Typhi and 18 *S*. Paratyphi A) *Salmonella enterica* blood isolates were included in the study, which were collected from the other three hospitals in Kolkata: 31 isolates from CMRI, 22 isolates from AGH and 15 isolates from AMRI. In other hospitals, median age of the enteric fever patients was 24.5 years (range, 1 to 63 years) of which 44 (64.7%) were male and 24 (35.3%) were female. Therefore a total of 77 *S*. Typhi and 25 *S*. Paratyphi A were characterized in this study.

### Distribution of antimicrobial resistance in the study isolates

**Figure 1:**
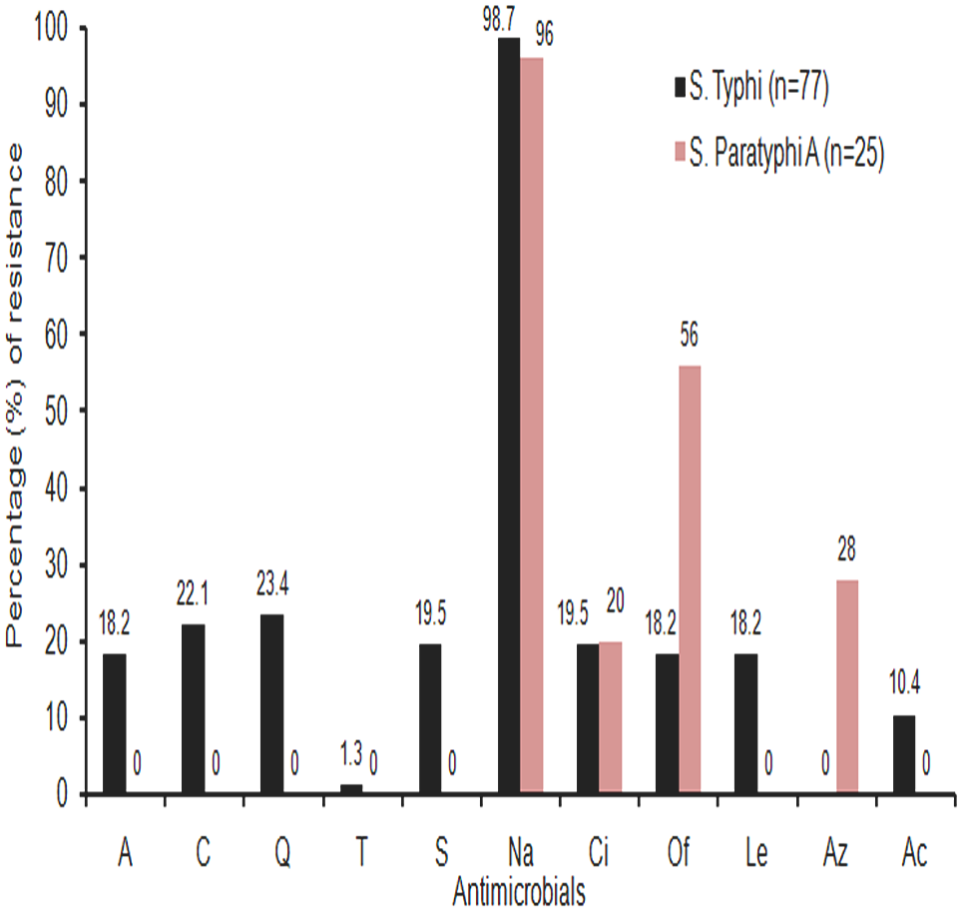
Percentage distribution of antimicrobial resistance in *S.* Typhi and *S.* Paratyphi A Kolkata isolates during 2009–2013. Interpretation was based on the MIC values of the antimicrobials. A, ampicillin; C, chloramphenicol; Q, co-trimoxazole; T, tetracycline; S, streptomycin; Na, nalidixic acid; Ci, ciprofloxacin; Of, ofloxacin; Le, levofloxacin; Az, azithromycin, Ac, amoxicillin/clavulanic acid.

All *Salmonella enterica* isolates were categorized into four groups based on the major AMR patterns: multidrug resistant (MDR), Nalidixic acid resistant (NaR), Decreased susceptibility to ciprofloxacin (DCS) and ciprofloxacin resistant (CiR) *Salmonella*. Year wise distribution of each AMR group in both the serovars are shown in Table 2. An increase in isolation of MDR *S*. Typhi was noticed from 2009 (13.6%) to 2013 (25%), but MDR isolates were not observed in *S*. Paratyphi A. Isolation of NaR isolates remained constant (>85%) in both the serovars throughout the study period and all NaR isolates were either CiR or showed DCS. Overall around 20% isolates of both the serovars were resistant to ciprofloxacin. Figure 1 shows the percentage distribution of antimicrobial resistance in *S*. Typhi and *S*. Paratyphi A Kolkata isolates during 2009–2013. It was observed that all *S*. Typhi isolates were susceptible to azithromycin and 7 (28%) *S*. Paratyphi A showed resistance to the drug. All study isolates were susceptible to third-generation cephalosporins (ceftriaxone, ceftazidime, cefotaxime) and aztreonam.

**Table 2:** Year wise distribution of antimicrobial resistant *S*. Typhi and *S.* Paratyphi A Kolkata isolates, 2009 to 2013.

Antimicrobial resistance Groups	*S*. Typhi				*S*. Paratyphi A			
	2009–2011 (n = 22)	2012 (n = 27)	2013 (n = 28)	Total (n = 77)	2009–2011 (n = 7)	2012 (n = 9)	2013 (n = 9)	Total (n = 25)
**MDR** a	3(13.6)	4(14.8)	7(25)	**14(18.2)**	0	0	0	**0**
**NaR** b	22(100)	26(96.3)	28(100)	**76(98.7)**	6(85.7)	9(100)	9(100)	**24(96)**
**DCS** c	16(72.7)	22(81.5)	23(82.1)	**61(79.2)**	5(71.4)	7(77.8)	7(77.8)	**19(76)**
**CiR** d	6(27.3)	4(14.8)	5(17.9)	**15(19.5)**	1(14.3)	2(22.2)	2(22.2)	**5(20)**

a MDR, multidrug resistant (resistance to ampicillin, chloramphenicol, co-trimoxazole with or without tetracycline).

b NaR, nalidixic acid resistant.

c DCS, decreased susceptibility to ciprofloxacin.

d CiR, ciprofloxacin resistant.

All data are in no. (%).

### MIC values and distribution

**Figure 2:**
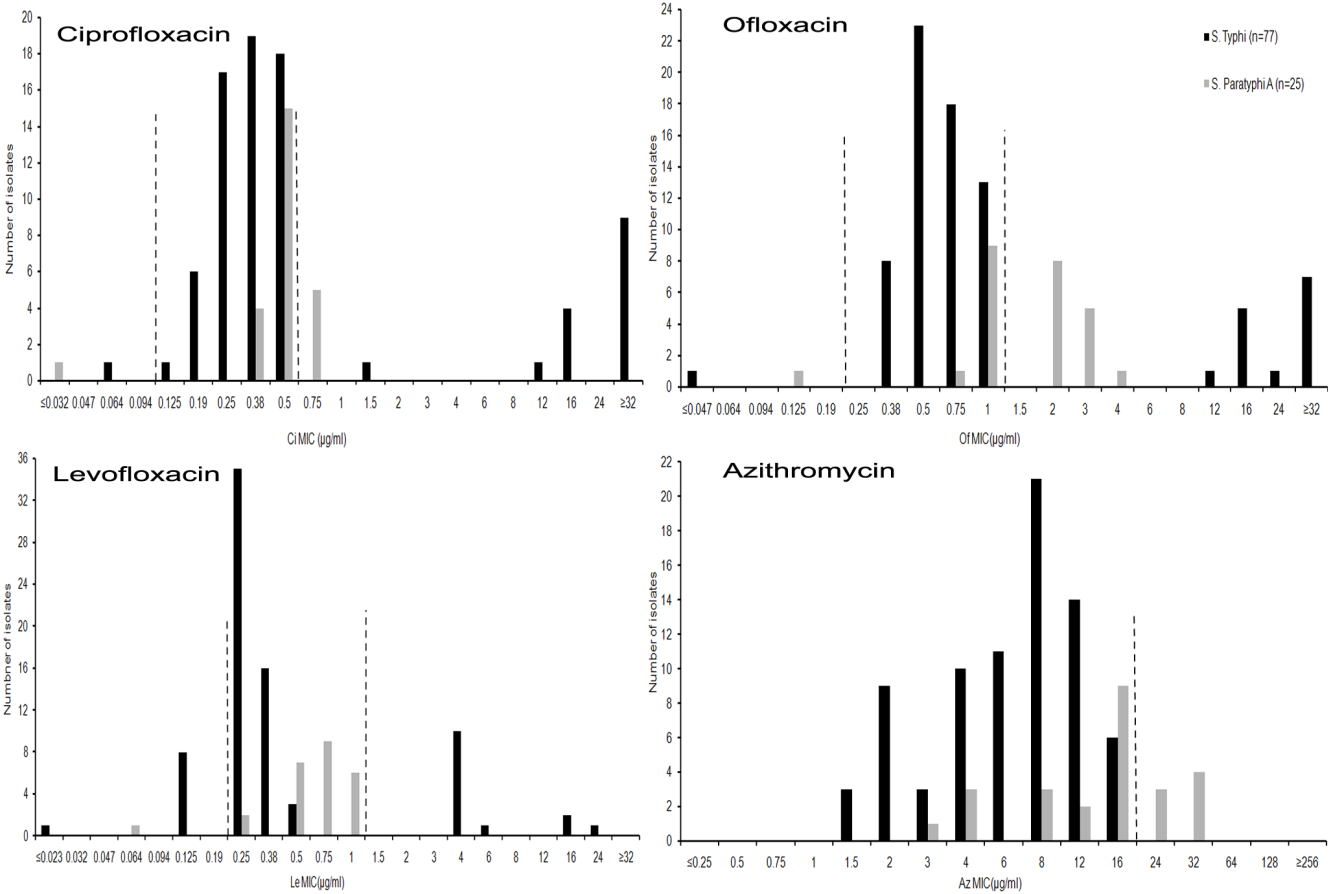
MIC distributions of fluoroquinolones and azithromycin in all *S*. Typhi and *S.* Paratyphi A Kolkata Isolates during 2009–2013. Interrupted lines denote MIC breakpoints of the antimicrobials for susceptible (left line) and resistant (Right Line) isolates. Isolates having MICs between two Interrupted lines showed reduced susceptibility. For azithromycin, single interrupted line indicates MIC breakpoint (>16 µg/ml) of resistance.

MIC Ranges, MIC_50_ and MIC_90_ of various antimicrobials are shown in Table 3. It is worth noting that the MIC_50_ of ciprofloxacin was 0.38 µg/ml for *S*. Typhi and 0.5 µg/ml for *S*. Paratyphi A, whereas MIC_90_ of ciprofloxacin was 32 µg/ml for *S*. Typhi and 0.75 µg/ml for *S*. Paratyphi A. MIC distributions of fluoroquinolones (FQs) like ciprofloxacin, ofloxacin and levofloxacin and azithromycin among the study isolates are shown in Figure 2. Interestingly, the majority of the study isolates of both the serovars showed MICs within reduced susceptibility zones of ciprofloxacin, ofloxacin and levofloxacin. Levofloxacin resistance was not observed in *S*. Paratyphi A. In *S*. Typhi isolates, MIC values of azithromycin were found below the resistance cutoff (>16 µg/ml) (Figure 2).

**Table 3:** MIC_50_ and MIC_90_ of *S*. Typhi (n = 77) and *S*. Paratyphi A (n = 25) isolates from Kolkata, 2009 to 2013.

Antimicrobial agents	MIC Range (µg/ml)		MIC_50_ (µg/ml)		MIC_90_ (µg/ml)		MIC (µg/ml) breakpoint for resistance^c^
	ST^a^	SPA^b^	ST^a^	SPA^b^	ST^a^	SPA^b^	
Ampicillin	0.094–≥256	1–3	0.5	1.5	≥256	3	**≥**32
Chloramphenicol	1.5–≥256	4–8	2	6	≥256	8	**≥**32
Co-trimoxazole	0.008–≥32	0.032–0.125	0.023	0.094	≥32	0.125	**≥**4
Tetracycline	1–16	1.5–4	1.5	3	3	4	**≥**16
Nalidixic acid	2–≥256	1.5–≥256	≥256	≥256	≥256	≥256	**≥**32
Ciprofloxacin	0.064–≥32	0.032–0.75	0.38	0.5	≥32	0.75	**≥**1
Ofloxacin	0.047–≥32	0.125–4	0.75	2	24	3	**≥**2
Levofloxacin	0.023–24	0.064–1	0.25	0.75	4	1	**≥**2
Ceftriaxone	0.032–0.19	0.064–0.19	0.064	0.125	0.125	0.19	**≥**4
Ceftazidime	0.064–0.38	0.19–0.75	0.19	0.38	0.38	0.5	**≥**16
Cefotaxime	0.023–0.125	0.125–0.25	0.064	0.19	0.094	0.19	**≥**4
Azithromycin	1.5–16	3–32	8	16	12	32	>16
Amoxicillin/clavulanic acid	0.25–≥256	0.75–3	0.5	1.5	32	1.5	**≥**32

a ST, *S.* Typhi.

b SPA, *S.* Paratyphi A.

c CLSI, 2013 guideline followed for all antimicrobials except azithromycin.

### Antimicrobial resistance profiles

Antimicrobial resistance profiles of the study isolates are shown in Table 4. NaR along with DCS was the most common resistance profile among both *S*. Typhi (58.4%) and *S*. Paratyphi A (40%). This was followed by resistance to nalidixic acid, ciprofloxacin, ofloxacin and levofloxacin in case of *S*. Typhi (16.9%) and resistance to nalidixic acid and ofloxacin in *S*. Paratyphi A (20%). All MDR *S*. Typhi (n = 14) isolates were simultaneously resistant to nalidixic acid and streptomycin and 8 of 14 showed resistance to amoxicillin/clavulanic acid (Table 4). One isolate in each serovar (*S*. Typhi and *S*. Paratyphi A) was susceptible to all the drugs tested. Strikingly different resistance profiles were observed among *S*. Typhi and *S*. Paratyphi A serovars with respect to the FQ resistance and multidrug resistance patterns (Table 4). Multidrug resistance pattern was observed in 18.2% of *S.* Typhi, but absent in *S*. Paratyphi A (Table 2). Levofloxacin resistance was common in *S.* Typhi (18.2%) and not in S. Paratyphi A. *S*. Paratyphi A showed resistance to azithromycin (28%) (Figure 1).

**Table 4:** Antimicrobial resistance profiles of *S.* Typhi and *S.* Paratyphi A Kolkata isolates from 2009–2013.

Antimicrobial resistance profile	*S*. Typhi (n = 77)	*S*. Paratyphi A (n = 25)
Na^a^	45(58.4)	10(40)
Na/Ci/Of/Le	13(16.9)	0
A/C/Q/S/Na/Ac^a^	7(9.1)	0
A/C/Q/S/Na^a^	6(7.8)	0
C/Q/Na^a^	3(3.9)	0
A/C/Q/S/Na/Ci/Ac	1(1.3)	0
T/Q/Na/Ci/Of/Le	1(1.3)	0
Na/Of^a^	0	5(20)
Na/Of/Az^a^	0	4(16)
Na/Ci/Of	0	3(12)
Na/Ci/Of/Az	0	2(8)

All data are in no. (%); Abbreviations used: A, ampicillin; C, chloramphenicol; Q, co-trimoxazole; T, tetracycline; S, streptomycin; Na, nalidixic acid; Ci, ciprofloxacin; Of, ofloxacin; Le, levofloxacin; Az, azithromycin; Ac, amoxicillin/clavulanic acid.

a associated with decreased susceptibility to ciprofloxacin (DCS); One isolate in each serovar was susceptible to all antimicrobials tested.

### Plasmid analysis and incompatibility testing

Of 14 MDR *S*. Typhi, 10 isolates harbored one heavy plasmid of about 180 kb, the remaining four isolates had no plasmid. A single plasmid of 180 kb was also present in three *S*. Typhi isolates resistant to chloramphenicol, co-trimoxazole and nalidixic acid. The heavy plasmids did not belong to any of the 18 major incompatibility groups [32]. One smaller plasmid of 50 kb size and IncN type was found in one *S*. Typhi isolate, which was resistant to tetracycline, co-trimoxazole, streptomycin, and quinolones (Table 5). Plasmid was not found in NaR *S*. Typhi isolates. Six *S*. Paratyphi A isolates harbored single plasmid (of either 2.5 or 3.5 kb size).

**Table 5:** Phenotypic and molecular characteristics of drug resistant *S*. Typhi (n = 18) isolates from Kolkata during 2009–2013.

Antimicrobialresistancegroup	SampleID no	R profile (n)	Plasmid (size)	Transfer ofresistancegenes byconjugation	Screening of resistance genes by PCR^a^								
					*bla*_TEM-1_	*cat*A	*sul*1	*sul*2	*dfr*A15	*aad*A1	*str*A	*str*B	Class 1integron^b^
MDR	BCR49	A/C/Q/S/Na	180 kb	−	+	+	+	+	−	−	+	+	+
	KOL34	(n = 6)	180 kb	−	+	+	+	+	−	−	+	+	+
	KOL61		180 kb	−	+	+	+	+	−	−	+	+	+
	KOL62		180 kb	−	+	+	+	+	−	−	+	+	+
	SP47		Absent	ND	+	+	+	+	−	−	+	+	+
	KOL39		Absent	ND	+	+	+	+	−	−	+	+	+
	BCR305	A/C/Q/S/Na/Ac	180 kb	−	+	+	+	+	−	−	+	+	+
	BCR342	(n = 7)	180 kb	−	+	+	+	+	−	−	+	+	+
	KOL17		180 kb	−	+	+	+	+	−	−	+	+	+
	KOL18		180 kb	−	+	+	+	+	−	−	+	+	+
	KOL38		180 kb	−	+	+	+	+	−	−	+	+	+
	KOL31		Absent	ND	+	+	+	+	−	−	+	+	+
	KOL37		Absent	ND	+	+	+	+	−	−	+	+	+
	BCR177	A/C/Q/S/Na/Ci/Ac	180 kb	−	+	+	+	+	−	−	+	+	+
Non MDR	KOL 36	C/Q/Na	180 kb	−	−	+	+	−	−	−	−	−	+
	KOL 42	(n = 3)	180 kb	−	−	+	+	−	−	−	−	−	+
	KOL 58		180 kb	−	−	+	+	−	−	−	−	−	+
	KOL 33^c^	T/Q/S/Na/Ci/Of/Le	50 kb	+	−	−	+	−	+	+	−	−	+^d^

Abbreviations used: MDR, multidrug resistant; ND, not done; A, ampicillin; C, chloramphenicol; Q, co-trimoxazole; T, tetracycline; S, streptomycin; Na, nalidixic acid; Ci, ciprofloxacin; Of, ofloxacin; Le, levofloxacin; Ac, amoxicillin/clavulanic acid.

a all isolates were negative for *bla*_OXA_, *bla*_SHV_ and *sul*3 genes by PCR.

b possessed *dfr*A7 gene cassette as determined by sequencing of 750 bp PCR amplicon.

c plasmid type: IncN; *tet*A was positive, but *tet*B was negative by PCR; transfer of *tet*A, *sul*1, *dfr*A15 and *aad*A1 genes by conjugation.

d gene cassette could not be determined by sequencing of 1.6 kb PCR amplicon.

### Transfer of antimicrobial resistance gene markers

The 180 kb plasmid of *S*. Typhi Kolkata isolates was not transferable, but the 50 kb plasmid present in one *S*. Typhi isolate (KOL 33) was transferable by conjugation experiment (Table 5). The respective *E. coli* transconjugant was resistant to tetracycline (MIC 16 µg/ml) and co-trimoxazole (MIC ≥32 µg/ml) but not to streptomycin (MIC 12 µg/ml) and quinolones by disc diffusion and E-test. The transconjugant was positive for *tet*A, *sul*1, *dfr*A15 and *aad*A1 genes by PCR.

### Determination of resistance and virulence gene markers by PCR

Table 5 shows distribution of AMR gene alleles and presence of integrons among ampicillin, chloramphenicol, co-trimoxazole and tetracycline resistant *S*. Typhi Kolkata isolates. Class 1 integrons present in *S*. Typhi study isolates had typical 3′ conserved segment comprising of *qac*EΔ1 and *sul*1 genes. A total of 55 *S*. Typhi and 21 *S*. Paratyphi A isolates were screened for virulence genes by PCR. The majority of *S*. Typhi and *S*. Paratyphi A isolates possessed all virulence markers tested indicating that these strains were likely to be virulent. The functional aspects of the genes, primer sequences, amplicon sizes and PCR positivity among study isolates are shown in Table 6.

**Table 6:** Virulence gene profiles by PCR of *S*. Typhi and *S*. Paratyphi A Kolkata isolates, 2009–2013.

Virulencegenes	Function	Oligonucleotide sequences (5′→3′)	PCR productsize (bp)	Positive by PCR		Reference
				*S.* Typhi(n = 55)	*S*. ParatyphiA (n = 21)	
*inv*A	Delivery of type IIIsecreted effectors	ACAGTGCTCGTTTACGACCTGAATAGACGACTGGTACTGATCGATAAT	500	55	21	[36]
*hil*A	Invasion proteintranscriptionalactivator	CCTTACGACGTATTCTGTCGGGCGATAATCCCTTCACGATA	207	55	21	This study
*ssa*Q	Type III secretorypathway protein	GAATAGCGAATGAAGAGCG TCC CATCGTGTTATCCTCTGTCAGC	677	45	21	[36]
*ssr*B	DNA-bindingresponse regulator intwo-componentregulatory system	GGTCTTGAGGTTTATAATGC CTG TCGTGTCAGCGTTTAATTCA	322	55	21	This study
*mgt*C	Intracellular survivaland growth in lowMg^2+^ environmentsduring systemicphase of the disease	ACGCACTAATGCGCTGGT GGCACGAATTTCTTTATAGCC	478	55	21	This study
*spi4*D	TypeI secretionprotein necessaryfor the secretion ofSpi4E	GAATAGAAGACAAAGCGAT CATC GCTTTGTCCACGCCTTTCATC	1231	53	19	[36]
*sop*B	Mediatesinflammation andfluid secretion inintestinal mucosa	ATGCTCAGGTCAAGCAGC ACCGTGGACATCCACAAA	214	55	21	This study
*sci*N	Type VI secretionlipoprotein	CGAATCACAGCGACTCGA TCATTTATCCCGTTCCGTAA	433	55	21	This study
*saf*B	Periplasmicfimbrialchaperone protein	TCCGTACTGGTGGTGACAT GGGATATACTCAAGCCCTGTAA	316	55	21	This study
*via*B	Vi polysaccharidebiosynthesis protein	TTGGCTCCGGCTTATTAGAA TGCAAACACATCAGCGTACA	460	55	ND	[23]
*stn*	Enterotoxin	TTGTCTCGCTATCACTGGCAACC ATTCGTAACCCGCTCTCGTCC	617	52	21	[36]
*plt*A	Delivery of CdtBfrom intracellularcompartment totarget cells	ACCTATCTGGAAACGCAAGGTGGTATACGTCAGCTCTTTGCCCTCGAT	343	55	21	This study

ND, not done.

### PFGE subtypes

**Figure 3:**
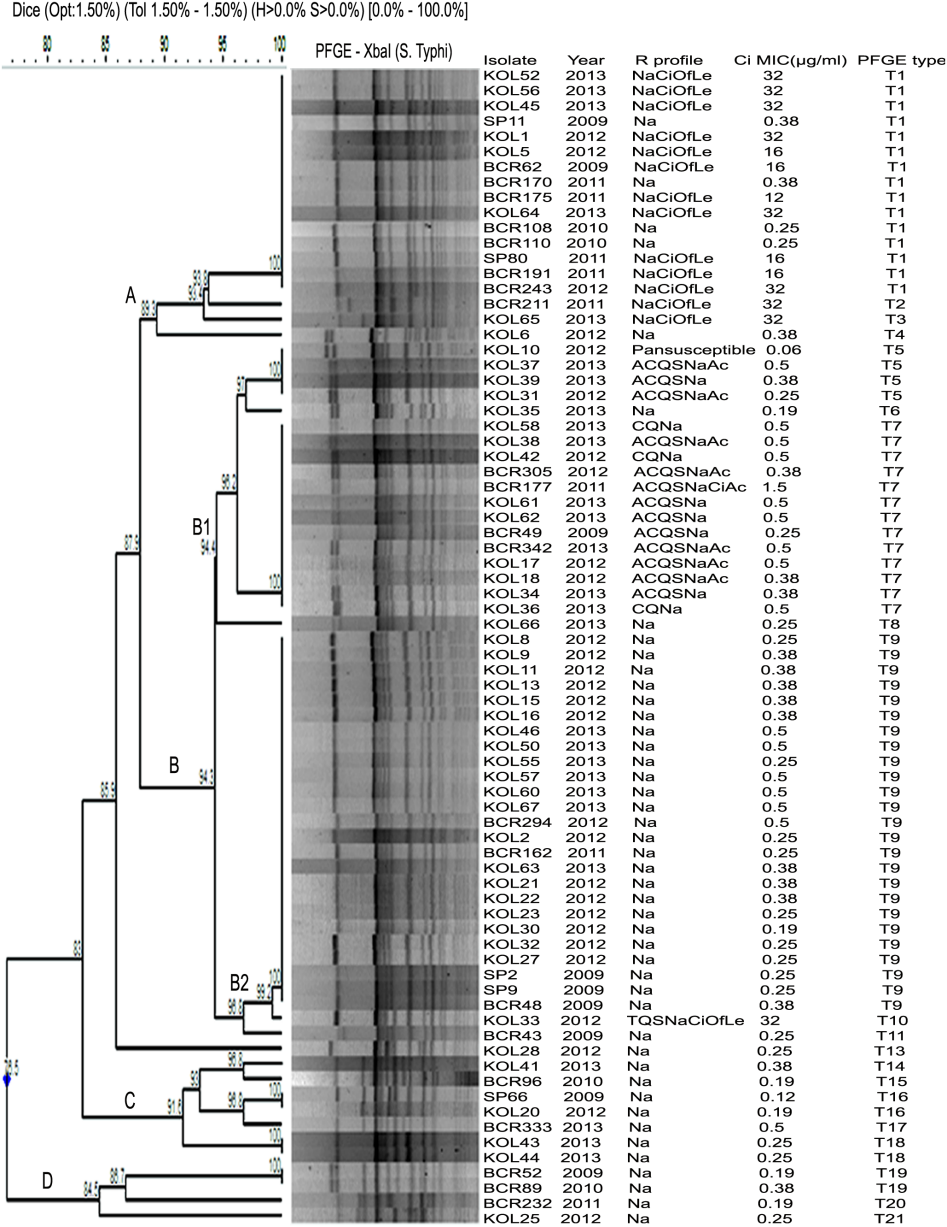
Dendrogram showing the cluster analysis of 76 *S.* Typhi isolates from Kolkata, India, 2009–2013, by *Xba*I-PFGE. Band comparison was performed by using the Dice coefficient with 1.5% optimization (Opt) and 1.5% position tolerance (Tol). Pan-susceptible, susceptible to all 17 drugs tested; A, ampicillin; Ac, amoxicillin/clavulanic acid; C, chloramphenicol; Q, co-trimoxazole; T, tetracycline; S, streptomycin; Na, nalidixic acid; Ci, ciprofloxacin; Of, ofloxacin; Le, levofloxacin.

Seventy six *S*. Typhi isolates generated 20 (T1 to T20) distinct PFGE types (pulsotypes). Total number of fragments generated ranged from 13 to 17 with sizes ranging from about 33 to 600 kb (Figure 3). All FQ resistant *S*. Typhi isolates, which were grouped under cluster A, 84.6% (11/13) were categorized under pulsotype T1, and other two isolates had distinct pulsotypes (T2 and T3). Cluster B showed two subclusters (B1 and B2). The MDR isolates (n = 13) belonged to subcluster B1 were grouped under two different pulsotypes T5 (n = 3) and T7 (n = 10), having high similarity co-efficient of 94.4%. Isolates of pulsotype T7 possessed one heavy plasmid of 180 kb size, whereas isolates of pulsotype T5 did not have any plasmid. *S*. Typhi (n = 3) with different AMR profile (resistant to chloramphenicol, co-trimoxazole and nalidixic acid) belonged to pulsotype T7. B2 subcluster comprised of mostly NaR isolates (n = 26). Maximum diversity was noted among NaR *S*. Typhi isolates, which were present in almost all clusters, e.g., cluster A (n = 5), cluster B1 (n = 2), cluster C (n = 7) and cluster D (n = 4). The pan-susceptible strain belonged to subcluster B1, pulsotype T5. An association between AMR types and PFGE types of *S*. Typhi was observed.

**Figure 4:**
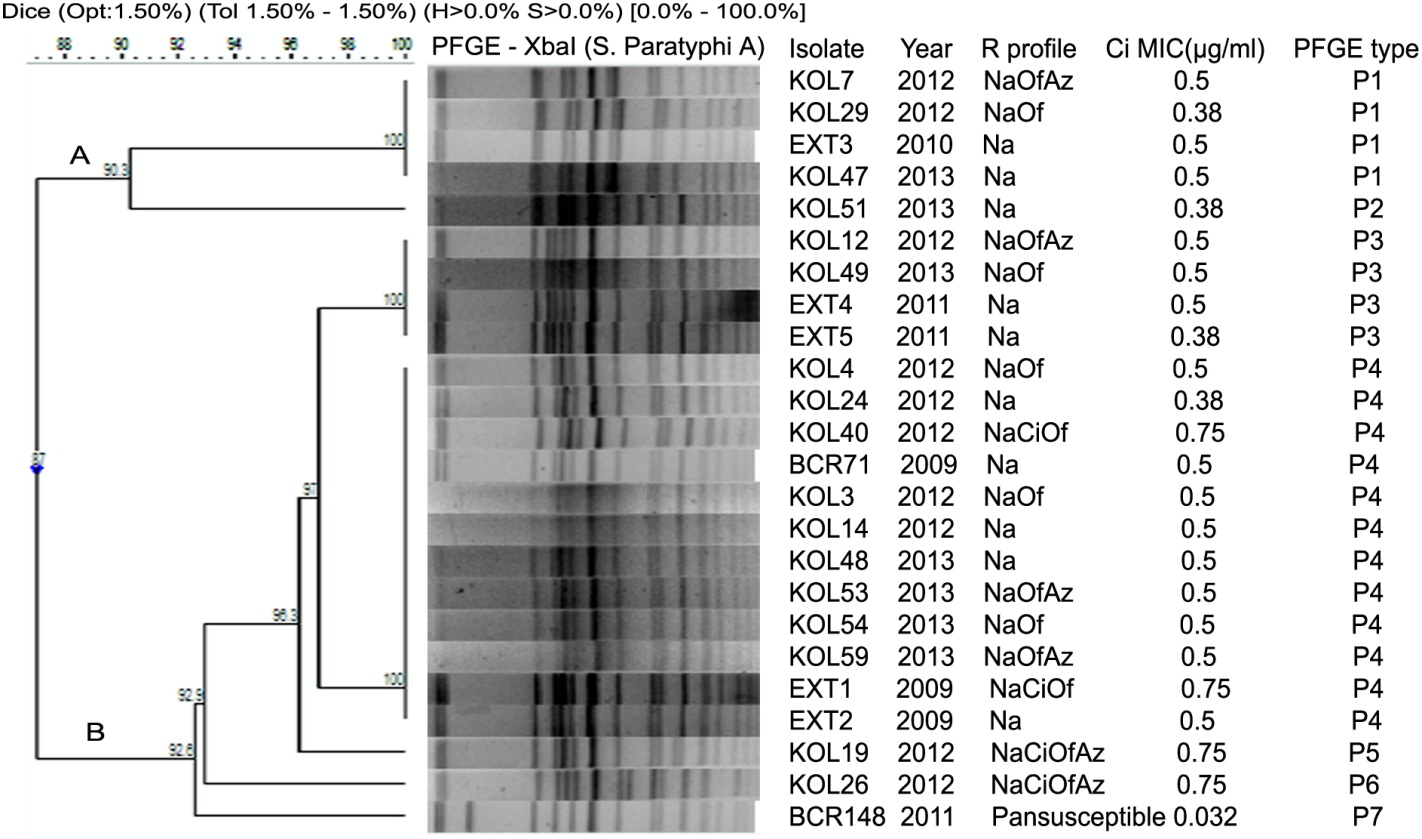
Dendrogram showing the cluster analysis of 24 *S.* Paratyphi A isolates from Kolkata, India, 2009–2013, by *Xba*I-PFGE. Band comparison was performed by using the Dice coefficient with 1.5% optimization (Opt) and 1.5% position tolerance (Tol). Pan-susceptible, susceptible to all 17 drugs tested; Na, nalidixic acid; Ci, ciprofloxacin; Of, ofloxacin; Az, azithromycin.

Two clusters (A and B) with 7 distinct pulsotypes (P1 to P7) were noted among 24 *S*. Paratyphi A (Figure 4). Like *S*. Typhi the total number of fragments ranged from 15 to 17 with sizes ranging from about 33 to 670 kb. The most common pulsotype was P4 (n = 12) followed by P1 (n = 4) and P3 (n = 4). Unlike *S*. Typhi, no association of resistance profiles and PFGE types was noted. One pan-susceptible isolate belonged to cluster B, subtype P7.

## Discussion

As enteric fever still continues to be an important public health problem in India and antibiotic therapy is the principal mode of treatment, we had undertaken this hospital based study to investigate the magnitude of this problem in Eastern part of India by isolating *Salmonella enterica* isolates from clinically diagnosed enteric fever cases during 2009–2013 and characterizing 77 *S*. Typhi and 25 *S*. Paratyphi A Kolkata isolates with respect to their AMR profiles, plasmid profiles, virulence profiles and PFGE pulsotypes.

In this prospective hospital based study, the ratio of isolation of *S*. Typhi (6.4%) and *S*. Paratyphi A (1.6%) was 4∶1, which was in accordance with the data reported from other parts of the country, where the ratio varied from 1.6∶1 to 3.7∶1 [3], [6], [9]. However, in recent years *S*. Paratyphi A has emerged as a predominant serovar over *S*. Typhi in India and China probably due to increased use of Vi polysaccharide vaccine [4], [5].

Increased occurrences of AMR in *S*. Typhi and *S*. Paratyphi A are of much concern worldwide in terms of non-availability of any rational treatment option for enteric fever. Observed gradual increase in isolation of MDR *S*. Typhi from 2009 (13.6%) to 2013 (25%) in this study was in tandem with the result of an earlier study from New Delhi which reported 34% isolation of MDR *S*. Typhi in 1999 and 66% in 2005 [39]. But other studies from Kolkata, Pondicherry, Delhi and Orissa showed decline in MDR *S*. Typhi isolation during late nineties [3], [9], [10], [11], [40]. No MDR *S*. Paratyphi A was isolated in this study, although increased isolation of MDR *S*. Paratyphi A ranging from 11.6% to 15.62% has been reported recently from other parts of the country [3], [4], [9]. An increased prevalence of MDR strains makes treatment with common drugs ineffective for typhoid.

Nalidixic acid resistance was observed in 98.7% *S*. Typhi and 96% *S*. Paratyphi A Kolkata isolates (Figure 1). Among this phenotype, most (>75%) of *S*. Typhi and *S*. Paratyphi A showed DCS (Table 4). A multi-centric study from India also reported increased isolation of NaR *S*. Typhi (83%) and *S*. Paratyphi A (93%), although DCS was not reported [6]. Similar observation was documented globally from other countries as well [14], [20]. In this study ciprofloxacin resistance was observed among 19.5% (15/77) *S*. Typhi (maximum MIC being ≥32 µg/ml) and 20% (5/25) *S*. Paratyphi A (maximum MIC being 0.75 µg/ml) Kolkata isolates (Figure 2), which was in sharp contrast with the result of an earlier community based study from Kolkata, where only a few CiR *S*. Typhi (2 isolates) [41] and *S*. Paratyphi A (1 isolate) (unpublished data) were recovered during 2003–2006. Studies from other parts of India documented variable isolation rate of CiR *S*. Typhi ranging from 8% to 21.4% during last decades [4], [10], [13]. High MICs of ciprofloxacin (≥512 µg/ml) was reported in *S*. Typhi and *S*. Paratyphi A in one North Indian study [42].

Among other FQs, ofloxacin resistance was more common in *S*. Paratyphi A (14/25, 56%) than *S*. Typhi (14/77, 18.2%) Kolkata isolates. Associated DCS was always present in both the serovars (Table 4). Levofloxacin resistance was observed only in *S*. Typhi (14/77, 18.2%) isolates, which was in contrast with the result of another study where both *S*. Paratyphi A (100%) and *S*. Typhi (92%) were levofloxacin resistant [43].

All *S*. Typhi and *S*. Paratyphi A isolates from Kolkata were susceptible to third-generation cephalosporins. *S*. Typhi, resistant to these antibiotics by producing ESBLs (CTX-M-15, SHV12), have been reported from other countries like Philippines, Nepal, Kuwait and U.A.E including India [3], [15], [16], [17]. Interestingly 10.4% *S*. Typhi Kolkata isolates showed high level resistance to amoxycillin/clavulanic acid (MIC ≥256 µg/ml), which was reported earlier [39].

Distribution of MICs of FQs for the study strains showed that majority of *S*. Typhi (88%) and *S*. Paratyphi A (>96%) isolates had MICs either in reduced susceptible zone or in resistant zone (Figure 2). In case of ciprofloxacin and ofloxacin resistant isolates, *S*. Typhi showed higher MICs of the antimicrobials than that shown by *S*. Paratyphi A.

Azithromycin is the empirical drug currently practiced for enteric fever but emergence of strains resistant to this antibiotic was a threat to the treatment with this drug [44]. In this study, 28% of azithromycin resistant *S*. Paratyphi A isolates showed MICs (MIC_90_ 32 µg/ml) well above the resistance cutoff (Table 3). *S*. Typhi study isolates showed MICs approaching towards the “epidemiological cutoff” for resistance (>16 µg/ml) due to frequent use of the drug (Figure 2). Azithromycin resistant *S*. Typhi and *S*. Paratyphi A have been reported from Delhi (MIC 24 µg/ml), Pakistan (MIC 64 µg/ml) and Cambodia (MIC 96 µg/ml) [18], [19], [20], [43]. For azithromycin, *in vitro* MICs may not always be consistent with *in vivo* test results [43]. Hence this drug should be used judiciously in enteric fever treatment.

The multidrug resistance gene markers in *S*. Typhi are usually located on mobile genetic elements like plasmids or integrons facilitating the transfer of resistance to other organisms [21], [22]. The conjugative 200 kb (approximately) plasmid associated with multidrug resistance and belonging to IncHI1 type was regarded as the globally dominant plasmid in *S*. Typhi [45]. Plasmid of incompatibility type IncHI1 in MDR *S*. Typhi was reported earlier from India [21], [46]. Plasmids of other incompatibility types like FII, FIA, P, B/O and HI1/FIIA were also reported from countries like Quetta, Pakistan, Malaysia and Kuwait [25]. However, interestingly in this study we observed that 71.4% (10/14) of MDR *S*. Typhi possessed single large (approximately 180 kb) plasmid which was non-conjugative and did not belong to any of the 18 major plasmid incompatibility groups including IncHI1 and 28.6% (4/14) MDR isolates did not possess any plasmid. Only one *S*. Typhi isolate (non-MDR) harboring a self-transmissible (conjugative) 50 kb plasmid of IncN type showed transfer of resistance to tetracycline and co-trimoxazole (Table 5). Similar transferable 50 kb plasmid in *S*. Typhi has been reported from Delhi [47]. *S*. Paratyphi A study isolates (24%, 6/25) harbored single plasmid of either 2.5 or 3.5 kb size. Single small plasmid of size range 1.7 to 3.8 MDa in *S*. Paratyphi A was reported from Japan earlier [48].

Ampicillin and chloramphenicol resistance in *S*. Typhi study isolates were encoded by *bla*_TEM-1_ and *cat*A genes respectively. Similar observation was reported earlier from India, Vietnam and China [4], [5], [9]. Class 1 integron with *dfr*A7 gene was responsible for trimethoprim resistance in *S*. Typhi isolates and sulphonamide resistance was encoded by *sul*1 and *sul*2 genes (Table 5). An earlier study from Vietnam confirmed our observation of presence of *sul*1, *sul*2 and *dfr*A7 genes in *S*. Typhi [9]. The single *S*. Typhi strain (KOL 33) resistant to tetracycline, co-trimoxazole and streptomycin possessed *tet*A, *sul*1, *dfr*A15, *aad*A1 and class 1 integron, which were transferable to the recipient strain (Table 5). Sequencing of variable region (1.6 kb) of class 1 integron in KOL 33 isolate was not possible, which would have determined resistance gene cassettes present in the isolate. Other associated mechanisms might also be involved for drug resistance in MDR *S.* Typhi. Exploring the mechanism of quinolone resistance among *Salmonella enterica* isolates is also important and yet to be investigated.

Not much variation was noted with respect to the distribution of virulence gene markers among *S*. Typhi and *S.* Paratyphi A Kolkata isolates (Table 6). All study *S*. Typhi isolates possessed *via*B gene, which is reported to be present in SPI7 locus, but *via*B negative *S*. Typhi was also reported from India [23]. Virulence genes like *inv*A, *ssa*Q, *spi4*D, *stn, hil*A, *mgt*C and *sop*B, were present in majority of the study isolates as was reported in other studies [23], [36]. The Kolkata isolates showed presence of other virulence genes like *ssr*B, *sci*N, *saf*B and *plt*A as well, although determination of actual contribution of the genes for disease pathogenesis in the study isolates was beyond the scope of this study.

PFGE was successfully used since 1994 and is currently the most useful method for subtyping the sporadic or epidemic isolates of different serovars of *Salmonella* [5], [24], [46], [49]. In this study, MDR, NaR and FQ resistant *Salmonella enterica* isolates were associated with distinct PFGE types of T7, T9 and T1 respectively (Figure 3).

MDR *S*. Typhi Kolkata isolates formed one cluster (B1), and most of the isolates with 180 kb plasmid belonged to one major pulsotype (T7) (Figure 3). Similar patterns of association between PFGE pulsotypes and AMR patterns of *S.* Typhi were observed by researchers from South India and Pakistan [26], [46]. Contrary to this study result, multiple pulsotypes were observed among MDR *S*. Typhi isolates in other countries like Malaysia, Pakistan, Bangladesh and Tajikistan [25], [50].

Nalidixic acid resistance was the most common phenotype and the NaR *S*. Typhi Kolkata isolates formed one major pulsotype (T9). Several other pulsotypes were also observed in this group, which was supported by a Vietnam study [49]. Le et al, suggested that due to increased therapeutic use of FQs, small scale genetic changes occurred among bacterial generations resulting in formation of diverse PFGE types among NaR *S*. Typhi clones [49].

The PFGE subtype of FQ resistant *S*. Typhi isolates were not studied extensively earlier. Interestingly, the present study showed the clonal nature of FQ resistant *S*. Typhi isolates forming one distinct pulsotype (T1) in cluster A (Figure 3) indicating spread of CiR *S*. Typhi in Kolkata from a single progenitor strain. Earlier one study in the US showed multiple PFGE patterns among CiR *S*. Typhi isolates. All these *S*. Typhi isolates were recovered from patients having history of travel to India [51].

*S*. Paratyphi A isolates were found to be more genetically homogeneous than *S*. Typhi isolates which was supported by earlier studies also [48]. An outbreak of paratyphoid fever in New Delhi was caused by two related (>80%) but distinct clones of *S*. Paratyphi A [52]. Another study suggested that a limited number of clones were found in *S*. Paratyphi A from Pakistan, India, Indonesia and Malaysia [27]. Similarly, the high proportion of *S*. Paratyphi A infection in China during 2002 to 2007 was due to the emergence of a single clone [5]. Although the association between PFGE pulsotypes and AMR patterns was not very obvious in *S*. Paratyphi A isolates (Figure 4), but >87% similarity coefficient among *S*. Paratyphi A Kolkata isolates suggested clonal expansion of the organism from common progenitor strain and possibilities of controlling the disease by use of suitable vaccines.

## Conclusions

*S*. Typhi and *S*. Paratyphi A were found as two predominant serovars in Kolkata from clinically suspected enteric fever cases. Increase in AMR including fluoroquinolone resistance among *S*. Typhi and *S*. Paratyphi A blood isolates has made selection of appropriate antimicrobials more difficult for successful treatment of the disease. All *S*. Typhi (100%) and majority of *S*. Paratyphi A (72%) study isolates were susceptible to azithromycin, suggesting azithromycin may be used with caution for empirical treatment of enteric fever. Interestingly, the observed association between the pulsotypes and resistance types of *S*. Typhi isolates from Kolkata might be useful in identifying the original source of infection followed by formulating strategies to control spread of the organism by appropriate interventions. Thus continuous monitoring of AMR and molecular subtypes of *Salmonella* blood isolates from endemic regions is recommended for better understanding of epidemiology of enteric fever.
